# *In vitro* investigation of head and neck cancer stem cell proportions and their changes following X-ray irradiation as a function of HPV status

**DOI:** 10.1371/journal.pone.0186186

**Published:** 2017-10-13

**Authors:** Paul Reid, Puthenparampil Wilson, Yanrui Li, Loredana G. Marcu, Alexander H. Staudacher, Michael P. Brown, Eva Bezak

**Affiliations:** 1 School of Health Sciences, University of South Australia, Adelaide, Australia; 2 Sansom Institute for Health Research, University of South Australia, Adelaide, Australia; 3 School of Engineering, University of South Australia, Adelaide, Australia; 4 Department of Medical Physics, Royal Adelaide Hospital, Adelaide, Australia; 5 Faculty of Science, University of Oradea, Oradea, Romania; 6 Translational Oncology Laboratory, Centre for Cancer Biology, SA Pathology, and University of South Australia, Adelaide, Australia; 7 School of Medicine, University of Adelaide, Adelaide, Australia; 8 Cancer Clinical Trials Unit, Royal Adelaide Hospital, Adelaide, Australia; 9 School of Physical Sciences, University of Adelaide, Adelaide, Australia; Università degli Studi della Campania "Luigi Vanvitelli", ITALY

## Abstract

**Introduction:**

Some head and neck squamous cell carcinomas (HNSCC) have a distinct aetiology, which depends on the presence of oncogenic human papilloma virus (HPV). Also, HNSCC contains cancer stem cells (CSCs) that have greater radioresistance and capacity to change replication dynamics in response to irradiation compared to non-clonogenic cells. Since there is limited data on CSCs in HNSCC as a function of HPV status, better understanding of their radiobiology may enable improved treatment outcome.

**Methods:**

Baseline and post-irradiation changes in CSC proportions were investigated by flow cytometry in a HPV-negative (UM-SCC-1) and a HPV-positive (UM-SCC-47) HNSCC cell line, using fluorescent staining with CD44/ALDH markers. CSC proportions in both irradiated and unirradiated cultures were compared for the two cell lines at various times post-irradiation. To assess repopulation of CSCs, untreated cultures were depleted of CD44+/ALDH+ cells and re-cultured for 3 weeks before flow cytometry analysis.

**Results:**

CSC proportions in untreated cell lines were 0.57% (UM-SCC-1) and 2.87% (UM-SCC-47). Untreated cell lines depleted of CD44+/ALDH+ repopulated this phenotype to a mean of 0.15% (UM-SCC-1) and 6.76% (UM-SCC-47). All UM-SCC-47 generations showed elevated CSC proportions after irradiation, with the most significant increase at 2 days post-irradiation. The highest elevation in UM-SCC-1 CSCs was observed at 1 day post-irradiation in the 2^nd^ generation and at 3 days after irradiation in the 3^rd^ generation. When measured after 10 days, only the 3^rd^ generation of UM-SCC-1 showed elevated CSCs.

**Conclusions:**

CSC proportions in both cell lines were elevated after exposure and varied with time post irradiation. UM-SCC-47 displayed significant plasticity in repopulating the CSC phenotype in depleted cultures, which was not seen in UM-SCC-1.

## Introduction

### Head and neck cancer: Aetiology and treatment challenges

Head and neck cancers comprise epithelial tumours of the mucosal linings of the oral and nasal cavities, the tongue, paranasal sinuses, salivary glands as well as the pharyngeal and laryngeal areas. Squamous cell carcinoma makes up around 90% of these cancers [1] which has a global incidence rate of approximately 680,000 new cases each year [2]. The survival rate for head and neck cancers is low and remains little changed over the last few decades, being around 50% at 5 years after diagnosis [3]. Metastatic disease is relatively uncommon but still impacts seriously on survival with locoregional recurrence of these tumours being the most frequent cause of mortality [4, 5].

Risk factors for head and neck cancers include tobacco and alcohol consumption and in countries across South East Asia and the Indian sub-continent, the chewing of betel quid [6, 7]. Of late, greater prominence is given the involvement of the human papilloma virus (HPV). In particular, HPV type 16 is shown to be a high risk subset of the virus and is implicated in oropharyngeal cancers (OPCs) where an increasing incidence is reported among young males in developed countries [8]. While rare, Fanconi anaemia, a recessive genetic disorder, is associated with a particularly aggressive form of head and neck cancer and an incidence rate around 800 times that of the normal population [9].

Head and neck squamous cell carcinoma (HNSCC) are typically aggressive cancers, often involving surrounding normal tissue. Management usually involves a multidisciplinary approach where radiotherapy is a principal intervention. The radiation dose is delivered using conventional or altered fractionation schedules and conformal treatment techniques, e.g. intensity modulated radiotherapy (IMRT), designed to minimise normal tissue complications while aiming for optimal tumour control [10].

### Cancer stem cell properties and their identification in HNSCC

HNSCCs contain complex heterogeneous populations where cells demonstrate varied phenotypes and sensitivities to chemotherapy and radiotherapy. A sub-population among these cells has attributes analogous to those of stem cells in normal tissue in that they can self-renew indefinitely and generate other more differentiated cells of the tumour population [11, 12]. These cells, known as cancer stem cells (CSCs), have shown themselves to be more radioresistant than other tumour cells as well as more effective in repairing radiation damage [13–15].

The proportion of CSCs in untreated tumours may typically be around 1–10% but this can vary greatly between cancer types, tumours of the same cancer type, and even within the same cancer cell line subjected to different treatments [16]. There is some evidence that higher CSC proportions in tumour populations correlate with a greater incidence of recurrence and poorer prognosis [17]. Thus, quantifying CSC proportions is important to understanding their behaviour and to optimise treatment planning [18–20]. Demonstrating this are reports that the proportion of CSCs within the tumour may increase not just from preferential survival, but also elevated self-renewal in response to therapeutic radiation and therefore during treatment [21]. CSCs can alter divisional dynamics by switching replication from asymmetrical (one daughter cell has the CSC phenotype while the other is non-CSC) to symmetrical division (both daughter cells are CSC phenotypes). This can rapidly increase their population, contributing to a potential trebling in their tumour proportion, and accelerating tumour repopulation [22, 23]. The composition of CSCs in tumour populations shows remarkable plasticity and the de-differentiation of non-CSCs into a stem cell state has also been reported in response to radiation [24, 25] demonstrating capacity among tumour cells to re-establish CSCs and facilitate tumour recurrence. These attributes, and the evidence of negative prognostic implications for tumours with higher CSC proportions [18, 26], demonstrate not only the critical need to target CSCs in treatment but also to understand their behaviours and radiobiological response. As yet, there is relatively little radiobiological data on CSCs in HNSCC and further investigation is required to better manage tumour control and the risk of recurrence.

Several different markers, both cell surface and functional, have been used to identify CSCs in tumour populations by studies of tumorigenicity using limiting dilution assays [27–30]. Cluster of differentiation 44 (CD44) is a cell surface protein and receptor for hyaluronic acid. Elevated levels of CD44 have been associated with CSCs in many different cancer studies including HNSCC [31, 32]. CD44 however, has been found to be broadly expressed in head and neck epithelium raising questions of its specificity [33]. Aldehyde dehydrogenase (ALDH), a metabolic enzyme, is also associated with CSCs by its elevated expression. ALDH expression has been found in a subset of the CD44+ population and therefore may be used to refine the selection of a putative CSC phenotype [34].

This study was conducted as a pilot, employing the novel construct of generational cultures of cell lines to test radiobiological responses among the clonogenic population of cell lines as a function of HPV status. Consequently, at this initial stage, representation of HPV status in HNSCC was limited to 1 cell line of each, representing some of the most common presentations of HNSCC and typically, presenting in the same clinic, that would be treated by the same protocol. *In vitro* experiments were conducted in order to identify and measure baseline CSC proportions by the CD44+/ALDH+ phenotype for the two untreated HNSCC cell lines. We also investigated the capacity of CSCs to repopulate in cultures sorted to be negative for the CD44+/ALDH+ phenotype. Finally, time-dependent changes in CSC proportions in surviving cell populations post X-ray irradiation were also investigated in post-irradiated cell generations of each cell line.

## Materials and methods

This experimental work investigated: a) baseline proportions of CSCs in two HNSCC cell lines; b) changes in CSC proportions following depletion by cell sorting; c) changes in CSC proportions post irradiation with 4 Gy X-ray. This dose was selected over the more conventional 2 Gy dose, used as a fractionated dose in HNSCC treatment, to provide a clear cellular response to a smaller number of subsequent exposures. Additionally, the temporal aspect of changes in CSC proportions in HNSCC cell lines after subsequent irradiations was investigated. The Human Research Ethics Committee of the University of South Australia has approved this study. Approval number 0000035359. This study only used commercially purchased laboratory cell lines.

### Cell line generations

**Fig 1:**
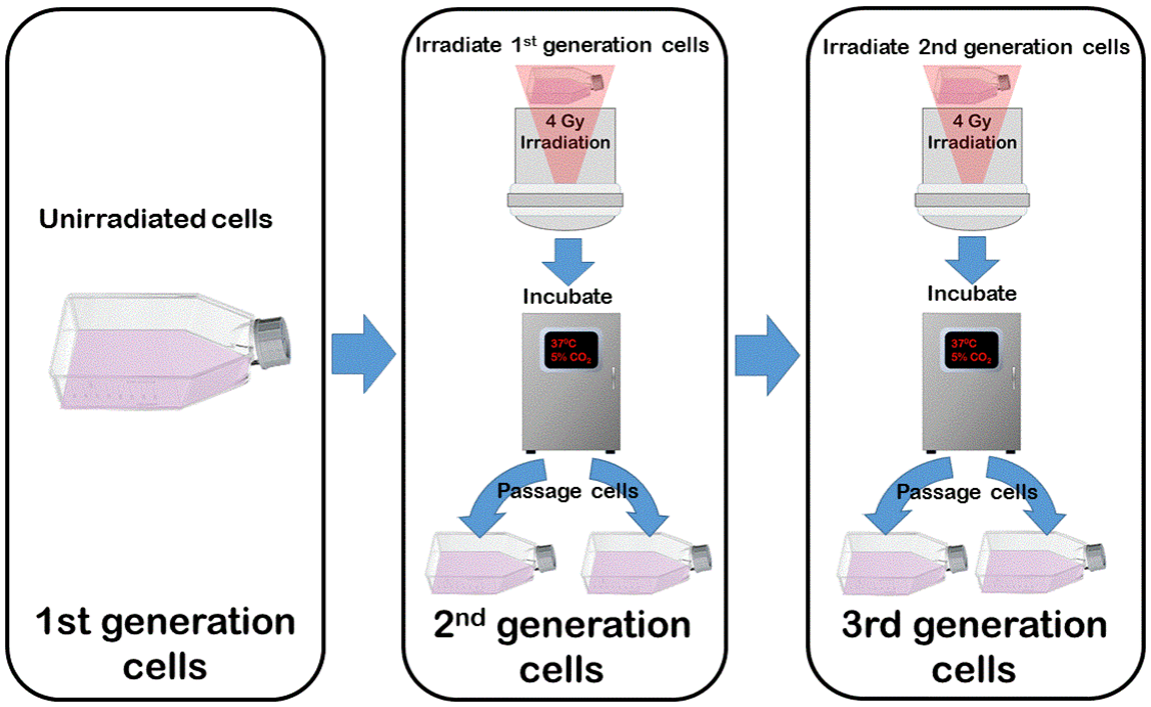
Process to establish subsequent generations of each cell line by re-irradiation and culturing.

In this study changes in the proportions of phenotypic populations, resulting from single or multiple irradiations, are called generational behaviour. The original unexposed culture of a cell line is termed the 1^st^ generation. When exposed to 4 Gy X-ray, then re-cultured and passaged, this then becomes the next generation (2^nd^ generation) and so forth for subsequent exposures. This provides for comparison within cell lines as to altered responses after repeated exposures where the unirradiated cells (1^st^ generation) are the control (Fig 1).

### Cell culture

Two head and neck cancer cell lines were use in this study. Both were purchased through Merck Millipore (Darmstadt Germany).

A. UM-SCC-1 was isolated from a recurrent squamous cell carcinoma in the mouth floor of a 73-year-old male. The cell line originated from the laboratory of Dr. Thomas Carey at the University of Michigan and is negative for the Human Papilloma Virus (HPV).

B. UM-SCC-47 is also a squamous cell carcinoma but derived from a primary tumour of the lateral tongue. This cell line also originated from the laboratory of Dr. Thomas Carey at the University of Michigan and is HPV positive for HPV type 16. Oncogenicity is conferred through the expression of viral oncoproteins E6 and E7 [35].

Cell lines were cultured in T75 flasks (Sigma-Aldrich^®^ Darmstadt DE) as a monolayer using RPMI 1640 medium (Sigma-Aldrich^®^ Darmstadt DE) supplemented with 10% foetal calf serum (FCS), 10 mM HEPES, 12.5 μg/ml penicillin and 16 μg/ml gentamycin. Cell flasks were incubated in a humidified atmosphere at 37°C containing 5% CO_2_ and passaged after reaching exponential growth prior to confluency. Both cell lines were tested for the presence of mycoplasma (biotool.com B3903, Madrid ES) and found to be negative.

### Cell staining

Cells were stained for both CD44 and ALDH to identify elevated expression of both markers among viable cells. In this study HNSCC CSCs were identified as CD44+/ALDH+ cells.

CD44 expression was measured using the fluorescent monoclonal antibody, Anti-Human/Mouse CD44 eFluor^®^ 450 (affymetrix eBioscience Santa Clara Ca.). Isotype control for this analysis was performed using Rat IgG2b K Isotype Control eFluor^®^ 450.

ALDEFLUOR^™^ (STEMCELLTM Technologies Vancouver BC.) was used as per manufacture’s protocol to determine ALDH activity. In the presence of ALDH, the substrate BODIPY-aminoacetaldehyde (BAAA), is converted to BODIPY-aminoacetate (BAA) proportional to ALDH present and is retained in cells. The intensity of the fluorescent expression in cells from this stain is proportional to the cellular ALDH activity. Negative control for this reaction was provided by diethylaminobenzaldehyde (DEAB) to cover background fluorescence at 1 μL in 100 μL cell suspension.

7-Aminoactinomycin D (7-AAD) (Thermo Fisher, Waltham Mass.) was used to distinguish between viable and dead cells.

Cells were initially stained with ALDEFLUOR^™^ and DEAB. After incubation at 37°C for 45 minutes, cells were then stained for CD44 and isotype and incubated at 4°C for 30 minutes before staining with 7-AAD, followed by flow cytometry analysis.

### Quantification of baseline CSC proportions

To determine baseline proportions of CSCs in unirradiated cultures, stained 1^st^ generation cells of both UM-SCC-1 and UM-SCC-47 were analysed in triplicate by flow cytometry (BD FACS Canto^™^ II, BD Biosciences, Franklin Lakes NJ.). As mentioned above, cells stained for DEAB and CD44 isotype act as negative controls for ALDH+ and CD44+, respectively, and were used to determine preliminary gating thresholds; additionally non-viable cells were excluded from the analysis.

### Cell sorting by CD44/ALDH

In this experiment, repopulation of the CSC phenotype has been investigated in both cell lines following CD44+/ALDH+ depletion by cell sorting. First generation UM-SCC-1 and UM-SCC-47 cells were stained and sorted into phenotypic groups by fluorescence activated cell sorting using a BD FACS Aria^™^ II (BD Biosciences, Franklin Lakes NJ.). Sorted cells from both cell lines, absent of the CD44+/ALDH+ CSC phenotype (CD44-/ALDH-), were re-cultured in T75 flasks for 3 weeks. Flow cytometry was then used to re-measure the same phenotype populations following re-culture and passaging, and the CSC proportions were compared with baseline measurements.

### CSC proportions in generational cell lines post 4 Gy irradiation

**Fig 2:**
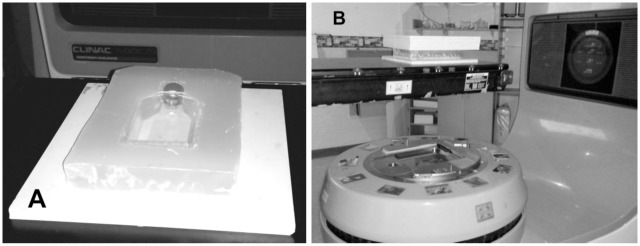
Irradiation setup for T75 flasks. A. T75 imbedded in wax atop 13 mm of solid water. B. Irradiations were performed from below with table top at isocentre.

To investigate generational changes in CSC proportions after irradiation, duplicate flasks of each generation (1^st^, 2^nd^ and 3^rd^) from both cell lines were irradiated with 4 Gy. One flask was used for flow cytometric analysis and the other re-cultured to grow the subsequent generation for later re-irradiation and analysis. Additionally, a flask of 1^st^ generation cells was unexposed and used as a sham irradiated control. Irradiation was performed using a 6 MV X-ray beam from a Varian 600C/D linear accelerator (Varian^®^ Medical System, Palo Alto, CA) at the Radiation Oncology Department of the Royal Adelaide Hospital. The linear accelerator was calibrated using IAEA TRS398 protocol [36] and the radiation dose output was checked on the day of irradiation with Daily QA 3^™^ device (Sun Nuclear, USA) before each radiation treatment. Flasks were positioned on top of 13 mm of solid water (RW3; PTW, Freiburg DE; ρ = 1.0459 g/cm^3^) directly above the isocentre of the beam and irradiated with the gantry at 180° to achieve an electronic equilibrium at the cell layer (see Fig 2). A 20 cm × 20 cm radiation field size was used and cell flasks were also encased in a paraffin block with a further 50 mm of solid water placed on top to achieve full scatter conditions.

Following irradiation, TrypLE^™^ (Thermo Fisher Scientific, Waltham Mass.) was used to disassociate adherent cells for flow cytometry analysis with optimal antigen retention [37]. Cells were centrifuged at 350 *g* for 5 minutes (Eppendorf 5810; Thermo Fisher Scientific, Waltham Mass.) before resuspension and replating then incubated for 24, 48 and 72 hours. At these time points, cells were again disassociated before counting by haemocytometer then aliquoted to 8 flow cytometry tubes, for staining of each generation and control, at 2 × 10^5^ cells per tube. Resulting CSC proportions were compared with baseline values.

### Temporal investigation of CSC proportion post irradiation

In order to investigate the temporal behaviour of any observed changes in CSC proportions, irradiated generations of both cell lines were also examined for CD44+/ALDH+ fractions by flow cytometry at 10 days post irradiation against matching controls. Controls as well as 2nd and 3rd generations were stained, as described above, to measure CSC phenotype proportions after this latent period for comparison with those of the same culture and treatment after only 3 days.

### Statistical analysis

Flow cytometry counting of CD44+/ALDH+ populations were analysed using FlowJo software (Tree Star, Ashland Ore.) to establish final gating and positive phenotype proportions in each sample. Results from FlowJo were analysed using Prism7.01 (GraphPad Software, Inc. La Jolla, CA). Values from triplicate analysis were averaged and reported as mean and standard error of the mean (SEM). Significance of difference for cell sorting and re-culture were tested by one-way ANOVA and Sidak’s test. For flow cytometry post 4 Gy irradiation, significance was calculated using two-way ANOVA and multiple comparisons between generations performed using Tukey’s test. Flow cytometry results post 10 days were tested by one-way ANOVA with Tukey’s tests for multiple comparisons. Significance was considered to be at p < 0.05 (* = p<0.05, ** = p<0.01, *** = p<0.001).

## Results

### Flow cytometry and cell sorting by CD44/ALDH

**Fig 3:**
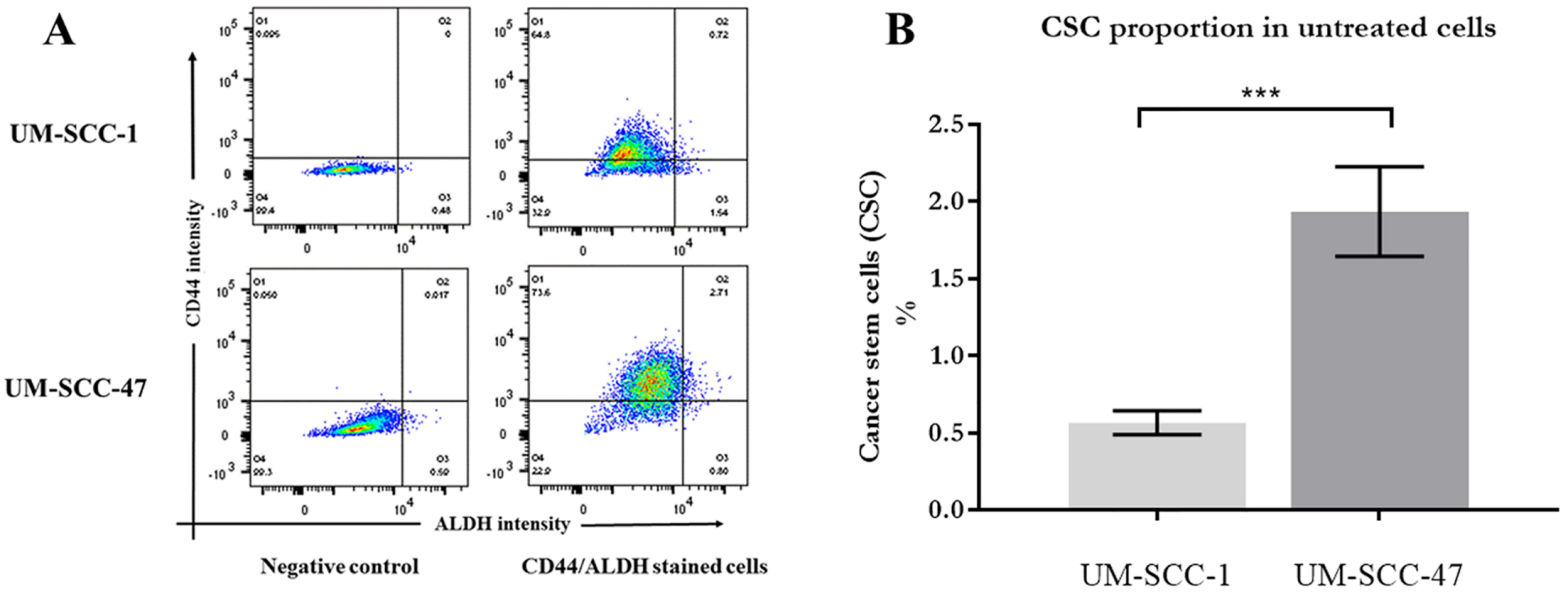
CSC proportions in untreated cells. A. Density plots from flow cytometry analysis of baseline proportions of cell phenotypes by CD44/ALDH expression. Upper right quadrants show percentages of cells positive for both CD44 and ALDH. B. Comparison of CSC percentage in untreated cell lines. The HPV positive UM-SCC-47 shows a significantly higher proportion of CSC by CD44+/ALDH+ phenotype than UM-SCC-1.

Triplicate analysis by flow cytometry of non-irradiated UM-SCC-47 cell cultures showed a mean population of CD44+/ALDH+ cells to be 2.87 ± 0.219, 5-fold that of the UM-SCC-1 population which was 0.57% ± 0.077 (see Fig 3).

### Repopulation responses following CSC depletion

**Fig 4:**
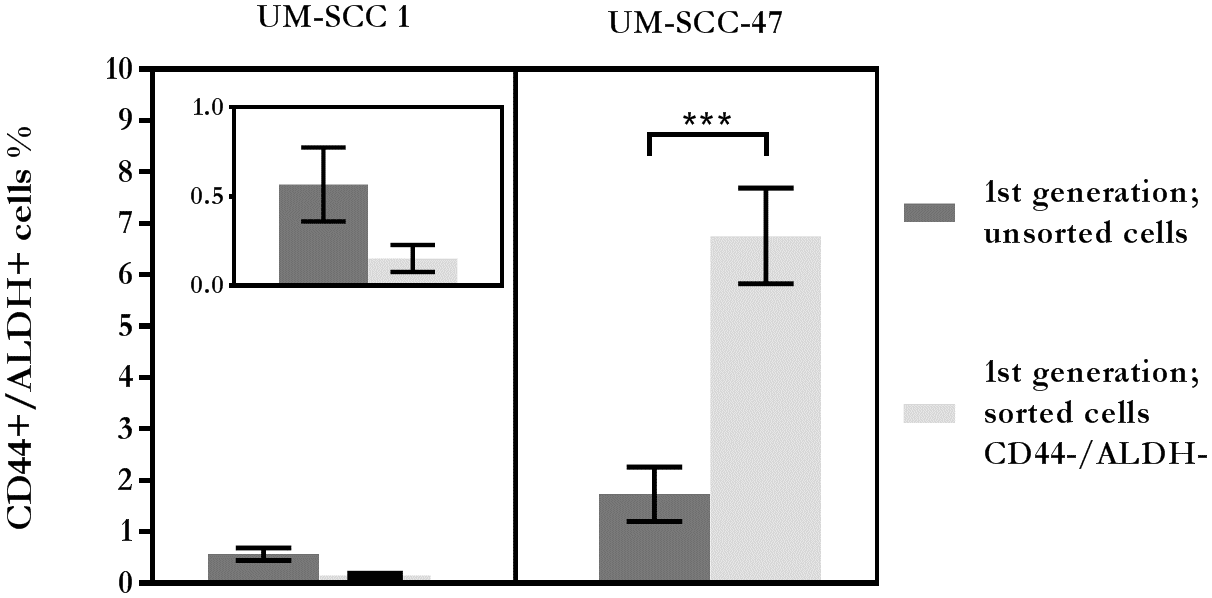
Repopulation of CD44+/ALDH+ cells in cell line cultures, sorted to be CD44-/ALDH-, compared to unsorted cells. Inset image is an enlarged scale of UM-SCC-1 to show error bars. (n = 3).

After cell sorting to deplete CD44+/ALDH+ cells, the repopulation of CD44+/ALDH+ cells was analysed by flow cytometry following a 21-day culture period (Fig 4). The mean population of CD44+/ALDH+ UM-SCC-1 cells was 0.15% ± 0.044, which was less than a third of the unsorted control. In clear distinction, vigorous repopulation of the CD44+/ALDH+ phenotype was observed among cultured cells of the sorted UM-SCC-47 population with a mean of 6.76% ± 0.932, which was greater than 2-fold increase of these cells compared to the unsorted control.

### CSC proportions post 4 Gy irradiation

**Fig 5:**
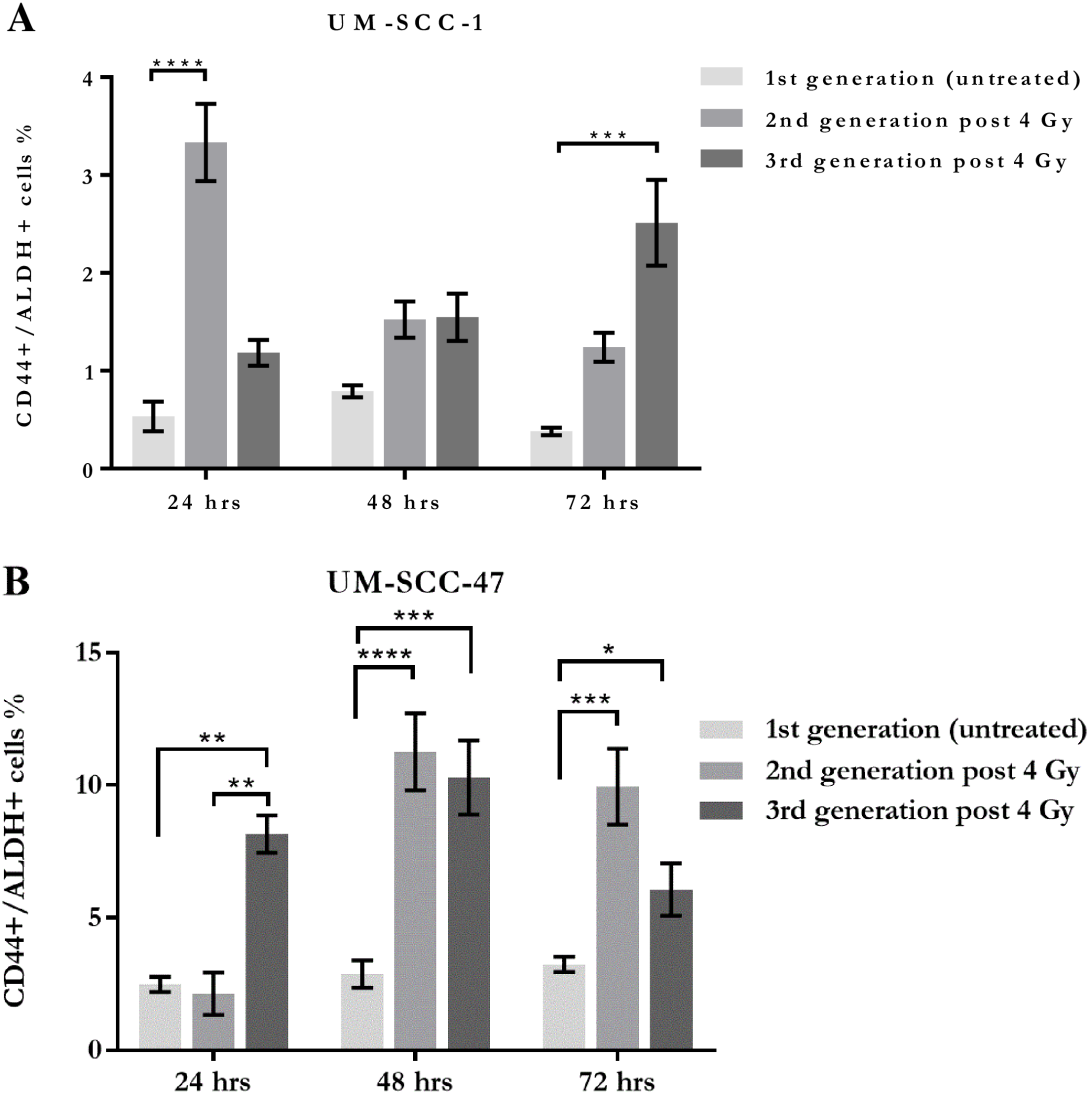
Summary of flow cytometry analysis. Percentages of CD44+/ALDH+ cells over 3 days, for A. UM-SCC-1 and B. UM-SCC-47, after 4 Gy X-ray irradiation, showing changes in the proportion of the putative CSC phenotype at each time point.

Both cell lines showed increases in the proportion of CD44+/ALDH+ cells in the surviving populations at each of the 3 time points for both generations of irradiated cells (Fig 5). The extent of these observed increases however, varied across the time intervals of 24, 48 and 72 hours, and also between the subsequent generations of each cell line.

The characterisation of CSC increases also varied between the cell lines. UM-SCC-1 showed early significant increases in the 2^nd^ generation with an approximately 6-fold increase, which diminished over the subsequent 3 days post-irradiation. A contrary response was observed in the 3^rd^ generation of UM-SCC-1 where the CSC population increased to a level around 6-fold that of the control at 72 hours. UM-SCC-47 showed the greatest elevation in CSC proportions at 48 hours for both the 2^nd^ and 3^rd^ generations where the observed increase was approximately 4-fold. UM-SCC-47 showed a greater number of significant results across the 3 time points for both exposed generations than UM-SCC-1 but less intergenerational difference.

### Temporal behaviour in CSC proportions at 10 days post irradiation

**Fig 6:**
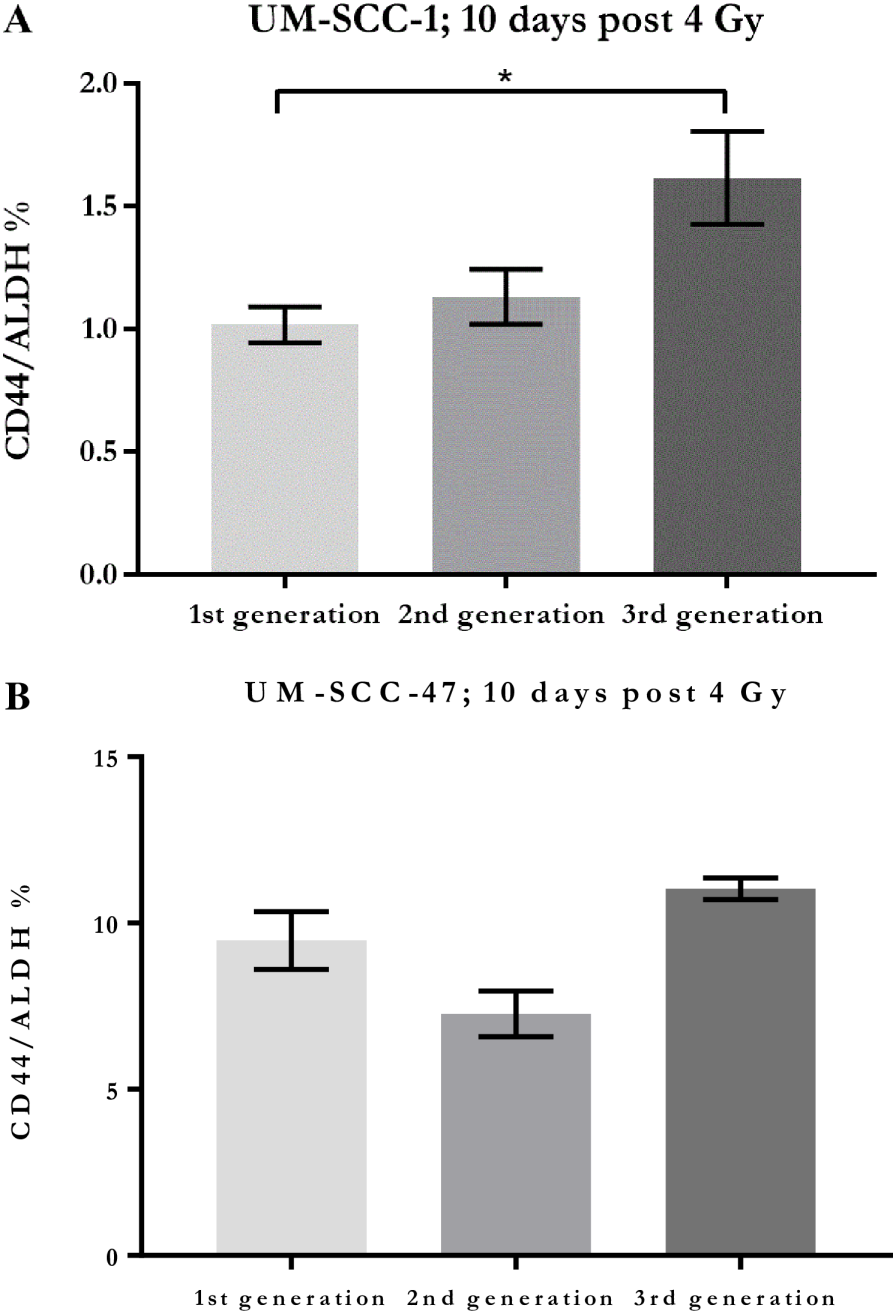
Comparative proportions of CD44+/ALDH+ cells. Proportions of CD44+/ALDH+ cells in surviving populations of each generation of A. UM-SCC-1 and B. UM-SCC-47 after 10 days (The 1^st^ generation is unirradiated control).

Comparative elevations in the CD44+/ALDH+ population, observed in UM-SCC-47 against the unirradiated control within 3 days post irradiation, were no longer significant after 10 days following parallel passaging of these generations. Of the UM-SCC-1 generations, only the 3^rd^ generation showed significance in elevation of CD44+/ALDH+ compared to the control after parallel passage over 10 days. The 2^nd^ generation showed only a slight, non-significant elevation (Fig 6).

## Discussion

Given the prominent role of radiotherapy in the treatment of HNSCC, planning for tumour control must aim to eliminate the cancers most refractory cells. Investigating the radiobiological behaviour of CSCs is crucial to understanding HNSCC treatment responses, resistance and tumour recurrence. A heightened radioresistance among CSCs is reported to be the result of their elevated endogenous capacity for antioxidant scavenging of the reactive oxygen species resulting from X-ray irradiation [11, 38]. More efficient repair of DNA damage is also demonstrated by CSCs allowing them to evade apoptosis and continue replication [14, 15]. Altered divisional dynamics among CSCs and a capacity for differentiation of tumour cells back to CSCs, facilitates accelerated repopulation and engenders recurrence after treatment which, as mentioned previously, is the leading cause of mortality in HNSCC. The CD44+/ALDH+ phenotype has been reported in literature as a marker to identify clonogenic CSCs in HNSCC [39–42]. Its elevated marker expression has been found to be indicative of a higher grade tumour and poor prognosis [43]. Given evidence from literature of CD44 and ALDH as phenotypic markers for CSCs in HNSCC, and their significance as prognostic factors by tumour proportion [18, 26, 40, 44] this study has used concomitant elevation in CD44 and ALDH for quantification of putative CSC proportions in treated and untreated cell line cultures.

In agreement with other studies of CSCs in HNSCC, we found that CSCs comprise a small proportion of the untreated cell lines [45]. Given the differing aetiology and better prognosis of HPV-positive HNSCC, we were interested to compare CSC proportions between these two cell lines. Surprisingly, we found the HPV-positive cell line (UM-SCC-47) had a significantly greater proportion of CSCs by the CD44+/ALDH+ phenotype. This result is in agreement with work by Zhang, Kumar [28] who also reported a higher proportion of CSCs among HPV-positive tumours (2.8%) compared to negative tumours (1.2%). These findings are at odds however with evidence from Vlashi, Chen [46] showing greater CSC proportions in HPV-negative tumour cell lines. It must be noted that their work identified CSCs using a different cellular functional marker, ZsGreen-cODC positive, which is a marker for proteasome activity.

Similarly, in contrast to Vlashi, Chen [46], we found evidence of a greater plasticity in the HPV-positive line. After sorting both cell lines to deplete CD44+/ALDH+ cells, HPV-positive UM-SCC-47 cells showed significant repopulation of CD44+/ALDH+ cells to 6.8%, which was a greater than 2-fold over the control. The proportion of cells bearing the CSC phenotype may increase if dedifferentiation among non-CSC cells returns these cells to a more primitive state of stemness [47, 48]. Our investigation examined the potential for both cell lines to repopulate the CD44+/ALDH+ fraction following its removal by sorting. We showed a distinct difference in the behaviour of the two cell lines. Sorted cells from UM-SCC-1 demonstrated negligible regeneration of CD44+/ALDH+ cells after 3 weeks whereas sorted UM-SCC-47 cells more than doubled the proportion of CD44+/ALDH+ cells compared to control. Here, UM-SCC-47 exhibited greater plasticity than UM-SCC-1 in being able to re-establish the CSC phenotype above the level of the control. The different repopulation abilities of the two cell lines, possibly via dedifferentiation, suggest that such heterogeneity may exist among other HNSCC cell lines and perhaps primary HNSCC tumours.

Evidence in literature that offers explanation for this observation is scant, but work on the induction of a stem cell state in somatic cells has involved mechanisms affected by the oncogenic factors that are characteristically different in HPV positive and negative HNSCC. Among human cancers, the *TP53* tumour suppressor gene is the most often mutated [49]. Mutated *TP53* characterises HPV-negative HNSCCs such as the UM-SCC-1 cell line. On the other hand, HPV-positive HNSCCs such as UM-SCC-47 typically have wild type *TP53*, which is instead inactivated by the E6 viral oncogene product [50–53]. Disruption of wild type TP53 function reportedly, may greatly potentiate dedifferentiation and favour the development of clonogenic populations [54, 55], which is facilitated by the HPV oncoprotein E6 and consistent with the results we have seen for the UM-SCC-47 and UM-SCC1 cell lines.

Visible regression in clinical HNSCC tumour volume during radiotherapy may misrepresent the state of therapeutic progress because CSCs respond to radiation by increasing their proportion among surviving cells. Even before the phenotypic identification of CSCs in solid tumours, a study by Withers, Taylor [56] examined the poorer prognostic outcomes in HNSCC where overall treatment time is prolonged. Its findings were that an accelerating regrowth in clonogenic cells could be active at a subclinical stage, even during treatment, and as the tumour mass was still regressing. Surviving CSC fractions are the basis for accelerated tumour repopulation and for this reason, the subclinical changes in CSC proportions cannot be overlooked as a risk for treatment failure.

The investigation of CSC proportions following 4 Gy irradiation, and subsequent re-irradiation, found variable and significant increases in the CSC proportions of surviving cell populations, in both cell lines. At some time-points across the three days after exposure, the proportion of CSCs had more than trebled, demonstrating the extent of phenotypic responsiveness apparent in these cell lines. Similar behaviour has been reported in other CSC studies for cancers such as breast and glioblastoma [57–59]. This may be indicative of a cancer’s potential to respond to radiation by repopulation with its most malignant phenotype.

Another point of difference between cell lines observed in this work, is the timing and extent of proportional increases in putative CSCs. UM-SCC-47 showed the most significant increases at 48 hours after irradiation for both exposed generations. UM-SCC-1 behaved differently with 2nd generation showing a significant increase early at 24 hours. Conversely, the 3rd generation showed increases in the CSC population later at 72 hours. Further to this, when these populations were measured again after 10 days, it was only the 3rd generation of UM-SCC-1 that showed any remaining significance in elevation of the CSC proportion. This implied a more persisting elevation in CSC fractions in UM-SCC-1, and possibly radioresistance, when subject to repeated exposures, unlike UM-SCC-47, which appears less phenotypically stable and more readily differentiated with repopulation.

To what extent this implies refractoriness in UM-SCC-1’s radiosensitivity warrants further study using an extended series of radiation treatments over a longer period of time. This may elucidate dynamics in the radiosensitivity of HNSCC that depends on tumour HPV status and may reflect biological responses that change during fractionated radiotherapy. Across the 3 days post-irradiation, the observed dynamic reapportionment of CSC numbers in response to irradiation may play a role in driving tumour repopulation. Alterations in CSC divisional dynamics, where replication switches from asymmetrical to symmetrical division, can accelerate tumour repopulation by rapidly increasing CSC numbers and hence their contribution to the generation of the total tumour cell number [22]. As mentioned above, the enhanced radioresistance and repair capacity of CSCs can affect their proportion simply by preferential survival, and dedifferentiation can recruit non-CSCs into this population [47, 48].

The temporal pattern of changes in CSC proportions, observed in the exposed generations 10 days after irradiation, demonstrate a distinction between the two cell lines. Significant elevations observed in the CD44+/ALDH+ population of the 2^nd^ and 3^rd^ generation of UM-SCC-47, within 3 days, were not seen when comparing between the parallel passaged generations after 10 days post irradiation. The 3^rd^ generation of UM-SCC-1 however, did demonstrate persisting significance in elevation of the CD44+/ALDH+ population compared to the parallel passaged control at 10 days.

The changes that we observed in the proportions of putative CD44+/ALDH+ CSCs, either after their irradiation or their depletion by cell sorting, suggest that CSCs may respond by altering the replicative programs that repopulate tumours, and by returning to quiescence in the subsequently re-established populations.

## Conclusion and future work

Analysis of putative CSCs in two HNSCC cell lines showed that although they occupied a small fraction of the total culture population, their proportion can be highly responsive to depletion from cell sorting or killing by 4 Gy irradiation. Both cell lines displayed significant increases in their CSC proportions in the 3 days post 4 Gy irradiation but this was varied across time points and between generations. The observed differences between the two cell lines in elevations of CSC proportions in each of the experiments raises questions about the contribution made by their HPV status. That a HPV-positive cell line shows higher baseline CSC levels and greater plasticity in repopulating a depleted culture suggests that HPV status may be an important determinant of functional CSC heterogeneity, and hence underscores the importance of further study.

These investigations are limited by the small sample size representing the HPV statuses in HNSCC. Future work needs to investigate heterogenic differences in the CSC subpopulation in terms of their HPV status to understand potential mechanisms for the better clinical outcomes seen in the HPV positive status and perhaps isolate that which makes the negative status more refractory.

Intergenerational differences exhibited in study of the UM-SCC-1 line also warrant further investigation. Extending the number of generations to examine if elevated proportions of CSCs become more persistent with time and number of radiation exposures may demonstrate the influence of this dependent variable on radiosensitivity and patterns of tumour recurrence. Any such finding would be relevant to the use of fractionated radiotherapy for HPV-negative HNSCC.

## Supporting information

S1 Fig Density plots for 3 generations of UM-SCC-1 and UM-SCC-47. Intensity of CD44 expression shown (y axis) against ALDH expression (x axis) for both cell lines at each time point. Upper right quadrants show percentages of cells positive for both CD44 and ALDH, which are putative CSCs.
(TIF)
