# Supplementary figures and images for: In vitro investigation of head and neck cancer stem cell proportions and their changes following X-ray irradiation as a function of HPV status
Source: PLoS One. 2017 Oct 13;12(10):e0186186. doi: 10.1371/journal.pone.0186186 (PMC5640219; doi:10.1371/journal.pone.0186186)

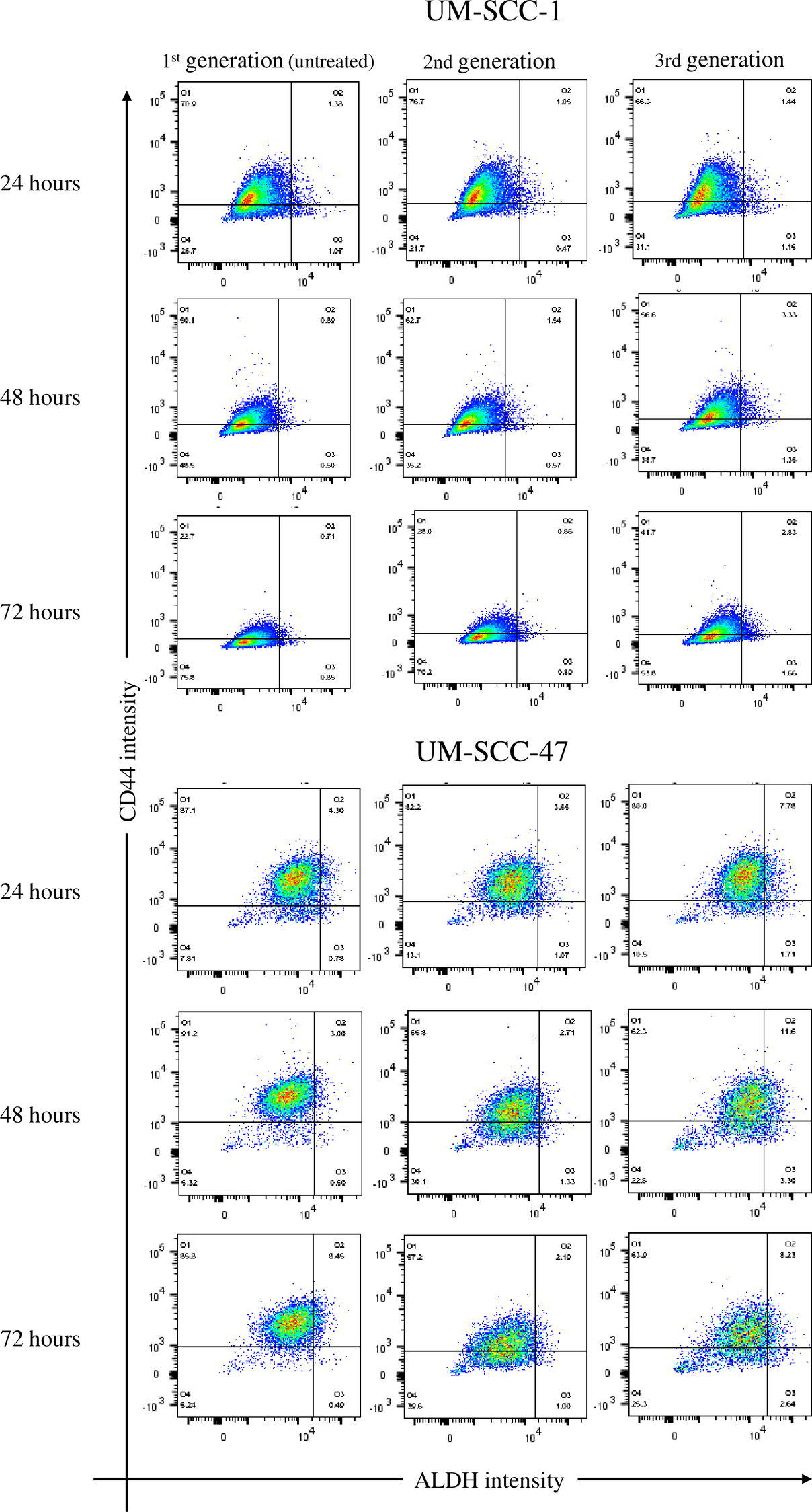

Supplement: S1 Fig — Intensity of CD44 expression shown (y axis) against ALDH expression (x axis) for both cell lines at each time point. Upper right quadrants show percentages of cells positive for both CD44 and ALDH, which are putative CSCs. (TIF) [file pone.0186186.s001.tif]
